# Supplementary material for: Identifying drivers of β-lactam/β-lactamase inhibitor resistance emergence and spread before their clinical deployment
Source: medRxiv. 2025 Jul 30:2025.07.29.25331838. Preprint. [Version 1] doi: 10.1101/2025.07.29.25331838 (PMC12324637; doi:10.1101/2025.07.29.25331838)
Supplement: 1 [file NIHPP2025.07.29.25331838V1-supplement-1.pdf]

SUPPLEMENTARY INFORMATION

Supplementary Table 1. Clade-specific differences in the presence of non-carbapenemase mechanisms of carbapenem resistance

Mechanism of interest	Overall (n=412)	Clade I (n=145)	Clade II (n=267)	p-value
**Carbapenem entry**				
ompK35 porin truncation at 25%	412 (100.0)	145 (100.0)	267 (100.0)	NA
ompK36 porin	131 (31.8)	129 (89.0)	2 (0.7)	<0.001
Cytosine-to-thymine transition at position 25	124 (30.1)	124 (85.5)	0 (0.0)	<0.001
Loop 3 insertion	119 (28.9)	119 (82.1)	0 (0.0)	<0.001
GD insertion	5 (1.2)	5 (3.4)	0 (0.0)	0.005
Non-synonymous mutation	22 (5.3)	13 (9.0)	9 (3.4)	0.029
Putative function-altering variants	20 (4.9)	13 (9.0)	7 (2.6)	0.007
Truncation	8 (1.9)	1 (0.7)	7 (2.6)	0.270
ompK36 intergenic region				
Any mutation	31 (7.5)	5 (3.4)	26 (9.7)	0.020
Insertion sequences	24 (5.8)	1 (0.7)	23 (8.6)	0.001
**Carbapenem efflux**				
acrAB-tolC efflux pump mutant	2 (0.5)	0 (0.0)	2 (0.7)	0.543
ramR efflux pump regulator putative function-altering variants	27 (6.6)	11 (7.6)	16 (6.0)	0.537
ramA efflux pump activator putative function-altering variants	10 (2.4)	4 (2.8)	6 (2.2)	0.746
**Carbapenem target modification**				
Non-synonymous mutations in penicillin-binding-proteins	9 (2.2)	3 (2.1)	6 (2.2)	1.000
Penicillin-binding protein-2	8 (1.9)	2 (1.4)	6 (2.2)	0.718
Penicillin-binding protein-4	2 (0.5)	2 (1.4)	0 (0.0)	0.123
**B-lactamases**				
$bla_{\text{CMY-2.v2}}$	12 (2.9)	9 (6.2)	3 (1.1)	0.005
$bla_{\text{CTX-M-14}}$	8 (1.9)	8 (5.5)	0 (0.0)	<0.001
$bla_{\text{CTX-M-15}}$	11 (2.7)	10 (6.9)	1 (0.4)	<0.001
$bla_{\text{KPC-2}}$	136 (33.0)	131 (90.3)	5 (1.9)	<0.001
$bla_{\text{KPC-3}}$	269 (65.3)	9 (6.2)	260 (97.4)	<0.001
$bla_{\text{KPC-5}}$	3 (0.7)	3 (2.1)	0 (0.0)	0.043
$bla_{\text{LAP-2}}$	1 (0.2)	0 (0.0)	1 (0.4)	1.000
$bla_{\text{OXA-1}}$	10 (2.4)	10 (6.9)	0 (0.0)	<0.001
$bla_{\text{OXA-9.v1}}$	154 (37.4)	1 (0.7)	153 (57.3)	<0.001
$bla_{\text{SHV-11}}$	313 (76.0)	135 (93.1)	178 (66.7)	<0.001
$bla_{\text{SHV-12}}$	30 (7.3)	2 (1.4)	28 (10.5)	0.001
$bla_{\text{TEM-104?}}$	6 (1.5)	6 (4.1)	0 (0.0)	0.002
$bla_{\text{TEM-104*?}}$	7 (1.7)	6 (4.1)	1 (0.4)	0.009
$bla_{\text{TEM-150*?}}$	6 (1.5)	0 (0.0)	6 (2.2)	0.095
$bla_{\text{TEM-1D.v1}}$	177 (43.0)	51 (35.2)	126 (47.2)	0.024
tn4401 **bla_{KPC} transposon isoform**				
tn4401a	143 (35.0)	138 (95.8)	5 (1.9)	<0.001
tn4401b	39 (9.5)	5 (3.5)	34 (12.8)	0.004
tn4401d	137 (33.5)	1 (0.7)	136 (51.3)	<0.001
tn4401_del_6920-7126	50 (12.2)	0 (0.0)	50 (18.9)	<0.001

tn4401_del_1-554_7008-7075	39 (9.5)	0 (0.0)	39 (14.7)	<0.001
tn4401_del_1-3391_6920-7126	1 (0.2)	0 (0.0)	1 (0.4)	1.000

Statistical significance was tested using the Chi-squared test for most variables. Fisher's exact test was performed for the following variables due to small event size: TD insertions in *ompk36* porin, acrAB-tolC efflux pump variant, *ramA*, *ramR*, penicillin-binding protein-2, penicillin-binding protein-4, *bla*$_{CTX\text{-}M14}$, *bla*$_{CTX\text{-}M\text{-}15}$, *bla*$_{KPC\text{-}5}$, *bla*$_{LAP\text{-}2}$, *bla*$_{OXA\text{-}1}$, *bla*$_{TEM\text{-}1}$, and *tn4401 del 1-3391 5920-7126*. For β-lactamase genes, ? indicates coverage < 100%, whereas *? indicates an incomplete match and coverage <100%.

Supplementary Table 2. Regression modeling to evaluate interaction between loop 3 insertions in *ompK36* porin with *bla*$_{KPC}$ copy number

	**Log$_2$ meropenem-vaborbactam minimum-inhibitory concentration**												**Log$_2$ imipenem-relebactam minimum-inhibitory concentration**											
**Predictors**	**Estimate**	**SE**	**CI**	**P-value**	**Estimate**	**SE**	**CI**	**P-value**	**Estimate**	**SE**	**CI**	**P-value**	**Estimate**	**SE**	**CI**	**P-value**	**Estimate**	**SE**	**CI**	**P-value**	**Estimate**	**SE**	**CI**	**P-value**
(Intercept)	-0.64	0.1	-0.83 – -0.45	**<0.001**	-1.68	0.09	-1.86 – -1.51	**<0.001**	-1.61	0.09	-1.78 – -1.43	**<0.001**	-1.12	0.06	-1.23 – -1.01	**<0.001**	-1.63	0.06	-1.74 – -1.52	**<0.001**	-1.61	0.06	-1.72 – -1.49	**<0.001**
Log$_2$ *bla*$_{KPC}$ copy number	-0.12	0.09	-0.30 – 0.05	0.169	0.3	0.07	0.16 – 0.44	**<0.001**	0.1	0.08	-0.06 – 0.27	0.205	0.02	0.05	-0.08 – 0.13	0.683	0.18	0.04	0.09 – 0.27	**<0.001**	0.12	0.05	0.01 – 0.23	**0.025**
Putative function altering variants in *ompK36*					2.4	0.31	1.79 – 3.02	**<0.001**	2.35	0.31	1.74 – 2.97	**<0.001**					2.52	0.2	2.13 – 2.92	**<0.001**	2.52	0.2	2.12 – 2.92	**<0.001**
Loop 3 insertions in *ompK36*					2.96	0.17	2.63 – 3.28	**<0.001**	3.19	0.17	2.86 – 3.52	**<0.001**					1.22	0.11	1.01 – 1.43	**<0.001**	1.3	0.11	1.08 – 1.51	**<0.001**
Log$_2$ bla$_{KPC}$ copy number * Putative function altering variants in *ompK36*									0.11	0.23	-0.34 – 0.56	0.627									0.01	0.15	-0.29 – 0.30	0.959
Log$_2$ bla$_{KPC}$ copy number * Loop 3 insertions in *ompK36*									0.89	0.17	0.55 – 1.23	**<0.001**									0.29	0.11	0.07 – 0.52	**0.009**
Observations	409				409				409				409				409				409			
R^2 / R^2 adjusted	0.005 / 0.002				0.461 / 0.457				0.495 / 0.489				0.000 / -0.002				0.387 / 0.383				0.398 / 0.390			
AIC	1695.218				1448.285				1425.753				1275.877				1079.84				1076.793			

The association between log$_2$ bla$_{KPC}$ copy number and log$_2$ minimum-inhibitory concentration (MIC) was assessed in bla$_{KPC}$-containing isolates. In simple linear regression, log$_2$ *bla*$_{KPC}$ copy number was not associated with increased log$_2$ MIC for MVB or IR. To account for the contribution of mutations to the *ompK36* porin, we adjusted for mutational status. While *ompK36* mutations exhibited strong independent associations with resistance in the additive model, a statistically significant association between log$_2$ *bla*$_{KPC}$ copy number and log$_2$ MIC for MVB and IR was also detected. Based on the marked increase in MIC associated with loop 3 insertions in *ompK36*, and their restriction to clade I, we hypothesized that loop 3 insertions were necessary for *bla*$_{KPC}$ copy number variation to influence MIC. Interaction modeling was performed to evaluate the relationship between loop 3 insertions and *bla*$_{KPC}$ copy number's influence on MICs. This modeling identified that the effect of *bla*$_{KPC}$ copy number was specific to isolates with loop 3 insertions, as confirmed by statistically significant interaction terms. **Abbreviations:** AIC, Akaike information criteria; CI, confidence interval; SE, standard error.

Supplementary Table 3. Unadjusted and adjusted analysis of risk factors for resistance to beta-lactam/beta-lactamase combinations

Variable	Overall (n=336)	Resistant (n=52)	Susceptible (n=284)	OR (95% CI)	OR p-value	aOR (95% CI)	aOR p-value
Age. 1 year unit	71.06 (14.66)	77.00 (11.72)	69.98 (14.90)	1.04 (1.02-1.07)	0.0017	1.04 (1.02-1.07)	0.0015
Female sex	40.99 (110.33)	41.81 (66.94)	40.85 (116.63)	1.00 (1.00-1.00)	0.9538		
Length of stay before culture, 1-day unit	160 (47.6)	28 (53.8)	132 (46.5)	1.34 (0.74-2.45)	0.3292		
Tracheostomy tube	227 (67.6)	33 (63.5)	194 (68.3)	0.81 (0.44-1.52)	0.4929		
Central venous catheter	187 (55.7)	25 (48.1)	162 (57.0)	0.70 (0.38-1.26)	0.2331		
Indwelling urinary catheter	183 (54.5)	29 (55.8)	154 (54.2)	1.06 (0.59-1.94)	0.8372		
Gastrostomy tube	121 (36.0)	16 (30.8)	105 (37.0)	0.76 (0.39-1.41)	0.3927		
Acute kidney injury	154 (45.8)	23 (44.2)	131 (46.1)	0.93 (0.51-1.68)	0.8009		
Chronic kidney disease	110 (32.7)	18 (34.6)	92 (32.4)	1.10 (0.58-2.04)	0.7537		
Ventilator-dependent respiratory failure	99 (29.5)	14 (26.9)	85 (29.9)	0.86 (0.43-1.64)	0.6622		
Underweight or malnutrition	84 (25.0)	7 (13.5)	77 (27.1)	0.42 (0.17-0.91)	0.0415	0.42 (0.16-0.93)	0.0466
Congestive heart failure	64 (19.0)	9 (17.3)	55 (19.4)	0.87 (0.38-1.82)	0.7283		
Decubitus ulcer stage 4+	67 (19.9)	7 (13.5)	60 (21.1)	0.58 (0.23-1.28)	0.2079		
Chronic bronchitis or chronic obstructive pulmonary disorder	60 (17.9)	13 (25.0)	47 (16.5)	1.68 (0.81-3.32)	0.1467		
Brain injury	63 (18.8)	11 (21.2)	52 (18.3)	1.20 (0.55-2.42)	0.6294		
Malignancy	42 (12.5)	6 (11.5)	36 (12.7)	0.90 (0.33-2.12)	0.8197		
Obesity	19 (5.7)	2 (3.8)	17 (6.0)	0.63 (0.10-2.28)	0.5425		
Carbapenem exposure	106 (31.5)	23 (44.2)	83 (29.2)	1.92 (1.04-3.51)	0.0342	1.95 (1.03-3.67)	0.0383
Amikacin exposure	41 (12.2)	8 (15.4)	33 (11.6)	1.38 (0.56-3.06)	0.4473		
Gentamicin exposure	12 (3.6)	2 (3.8)	10 (3.5)	1.10 (0.17-4.32)	0.9076		
Tobramycin exposure	21 (6.2)	5 (9.6)	16 (5.6)	1.78 (0.56-4.80)	0.2813		
Aztreonam exposure	10 (3.0)	0 (0.0)	10 (3.5)	0.00 (NA-Infinity)	0.9843		
Sulfamethoxazole-trimethoprim exposure	11 (3.3)	2 (3.8)	9 (3.2)	1.22 (0.18-4.92)	0.8011		
Cephalosporin exposure	83 (24.7)	20 (38.5)	63 (22.2)	2.19 (1.16-4.07)	0.0138	2.13 (1.10-4.07)	0.0232
Fluoroquinolone exposure	59 (17.6)	7 (13.5)	52 (18.3)	0.69 (0.27-1.54)	0.4003		
Daptomycin exposure	14 (4.2)	5 (9.6)	9 (3.2)	3.25 (0.96-9.84)	0.042		
Metronidazole exposure	62 (18.5)	10 (19.2)	52 (18.3)	1.06 (0.48-2.18)	0.875		
Linezolid exposure	48 (14.3)	6 (11.5)	42 (14.8)	0.75 (0.27-1.75)	0.5392		
Polymyxin and/or colistin exposure	38 (11.3)	6 (11.5)	32 (11.3)	1.03 (0.37-2.44)	0.9548		
Tigecycline exposure	47 (14.0)	11 (21.2)	36 (12.7)	1.85 (0.84-3.83)	0.1093		
Vancomycin exposure	143 (42.6)	21 (40.4)	122 (43.0)	0.90 (0.49-1.63)	0.7302		
Piperacillin/Tazobactam exposure	45 (13.4)	5 (9.6)	40 (14.1)	0.65 (0.22-1.59)	0.3875		

Abbreviations: aOR, adjusted odds ratio; CI, confidence interval; OR, odds ratio

Supplementary Table 4. Unadjusted and adjusted analysis of risk factors for non-carbapenemase mechanisms of resistance to beta-lactam/beta-lactamase combinations

Variable	Overall (n=317)	Resistant (n=34)	Susceptible (n=284)	OR (95% CI)	OR p-value	aOR (95% CI)	aOR p-value
Age. 1 year unit	70.75 (14.84)	77.20 (12.84)	69.98 (14.90)	1.04 (1.01-1.07)	0.0079	1.04 (1.01-1.07)	0.0112
Female sex	40.46 (110.78)	37.26 (35.17)	40.85 (116.63)	1.00 (0.99-1.00)	0.8591		
Length of stay before culture, 1-day unit	149 (46.9)	17 (50.0)	132 (46.5)	1.15 (0.56-2.36)	0.6976		
Tracheostomy tube	217 (68.2)	23 (67.6)	194 (68.3)	0.97 (0.46-2.15)	0.9375		
Central venous catheter	178 (56.0)	16 (47.1)	162 (57.0)	0.67 (0.32-1.37)	0.2701		
Indwelling urinary catheter	170 (53.5)	16 (47.1)	154 (54.2)	0.75 (0.36-1.53)	0.4296		
Gastrostomy tube	114 (35.8)	9 (26.5)	105 (37.0)	0.61 (0.26-1.32)	0.2311		
Acute kidney injury	143 (45.0)	12 (35.3)	131 (46.1)	0.64 (0.30-1.32)	0.2331		
Chronic kidney disease	103 (32.4)	11 (32.4)	92 (32.4)	1.00 (0.45-2.09)	0.9961		
Ventilator-dependent respiratory failure	93 (29.2)	8 (23.5)	85 (29.9)	0.72 (0.29-1.59)	0.4398		
Underweight or malnutrition	81 (25.5)	4 (11.8)	77 (27.1)	0.36 (0.10-0.95)	0.0615	0.40 (0.11-1.09)	0.1012
Congestive heart failure	59 (18.6)	4 (11.8)	55 (19.4)	0.56 (0.16-1.48)	0.2873		
Decubitus ulcer stage 4+	64 (20.1)	4 (11.8)	60 (21.1)	0.50 (0.14-1.32)	0.2061		
Chronic bronchitis or chronic obstructive pulmonary disorder	53 (16.7)	6 (17.6)	47 (16.5)	1.08 (0.39-2.60)	0.8711		
Brain injury	58 (18.2)	6 (17.6)	52 (18.3)	0.96 (0.34-2.28)	0.9247		
Malignancy	39 (12.3)	3 (8.8)	36 (12.7)	0.67 (0.15-1.99)	0.5201		
Obesity	18 (5.7)	1 (2.9)	17 (6.0)	0.48 (0.03-2.44)	0.4776		
Carbapenem exposure	100 (31.4)	17 (50.0)	83 (29.2)	2.42 (1.17-5.00)	0.0159	3.41 (1.52-7.77)	0.0031
Amikacin exposure	38 (11.9)	5 (14.7)	33 (11.6)	1.31 (0.42-3.37)	0.601		
Gentamicin exposure	12 (3.8)	2 (5.9)	10 (3.5)	1.71 (0.26-6.87)	0.4996		
Tobramycin exposure	20 (6.3)	4 (11.8)	16 (5.6)	2.23 (0.61-6.57)	0.1741		
Aztreonam exposure	10 (3.1)	0 (0.0)	10 (3.5)	0.00 (NA-Infinity)	0.9901		
Sulfamethoxazole-trimethoprim exposure	10 (3.1)	1 (2.9)	9 (3.2)	0.93 (0.05-5.16)	0.9427		
Cephalosporin exposure	76 (23.9)	13 (38.2)	63 (22.2)	2.17 (1.01-4.54)	0.0417	2.63 (1.10-6.26)	0.0281
Fluoroquinolone exposure	57 (17.9)	5 (14.7)	52 (18.3)	0.77 (0.25-1.93)	0.6055		
Daptomycin exposure	12 (3.8)	3 (8.8)	9 (3.2)	2.96 (0.63-10.52)	0.1177		
Metronidazole exposure	57 (17.9)	5 (14.7)	52 (18.3)	0.77 (0.25-1.93)	0.6055		
Linezolid exposure	44 (13.8)	2 (5.9)	42 (14.8)	0.36 (0.06-1.25)	0.172	0.26 (0.04-0.99)	0.0878
Polymyxin and/or colistin exposure	37 (11.6)	5 (14.7)	32 (11.3)	1.36 (0.44-3.50)	0.5559		
Tigecycline exposure	45 (14.2)	9 (26.5)	36 (12.7)	2.48 (1.03-5.58)	0.0337		
Vancomycin exposure	135 (42.5)	13 (38.2)	122 (43.0)	0.82 (0.39-1.69)	0.5989	0.46 (0.18-1.11)	0.0914
Piperacillin/Tazobactam exposure	43 (13.5)	3 (8.8)	40 (14.1)	0.59 (0.14-1.76)	0.4015		

Abbreviations: aOR, adjusted odds ratio; CI, confidence interval; OR, odds ratio.

Supplementary Table 5. Unadjusted and adjusted analysis of risk factors for plasmid-mediated mechanisms of resistance to beta-lactam/beta-lactamase combinations

Variable	Overall (n=302)	Resistant (n=18)	Susceptible (n=284)	OR (95% CI)	OR p-value	aOR (95% CI)	aOR p-value
Age. 1 year unit	70.37 (14.71)	76.63 (9.58)	69.98 (14.90)	1.04 (1.00-1.08)	0.0633	1.04 (1.00-1.08)	0.0771
Female sex	41.41 (115.80)	50.39 (104.52)	40.85 (116.63)	1.00 (0.99-1.00)	0.7389		
Length of stay before culture, 1-day unit	143 (47.4)	11 (61.1)	132 (46.5)	1.81 (0.69-5.04)	0.2336		
Tracheostomy tube	204 (67.5)	10 (55.6)	194 (68.3)	0.58 (0.22-1.57)	0.2673		
Central venous catheter	171 (56.6)	9 (50.0)	162 (57.0)	0.75 (0.29-1.98)	0.5599		
Indwelling urinary catheter	167 (55.3)	13 (72.2)	154 (54.2)	2.19 (0.80-6.99)	0.1451		
Gastrostomy tube	112 (37.1)	7 (38.9)	105 (37.0)	1.08 (0.39-2.84)	0.8703		
Acute kidney injury	142 (47.0)	11 (61.1)	131 (46.1)	1.84 (0.70-5.11)	0.2226		
Chronic kidney disease	99 (32.8)	7 (38.9)	92 (32.4)	1.33 (0.48-3.48)	0.5703		
Ventilator-dependent respiratory failure	91 (30.1)	6 (33.3)	85 (29.9)	1.17 (0.40-3.12)	0.7604		
Underweight or malnutrition	80 (26.5)	3 (16.7)	77 (27.1)	0.54 (0.12-1.68)	0.3371		
Congestive heart failure	60 (19.9)	5 (27.8)	55 (19.4)	1.60 (0.50-4.44)	0.3895		
Decubitus ulcer stage 4+	63 (20.9)	3 (16.7)	60 (21.1)	0.75 (0.17-2.35)	0.6526		
Chronic bronchitis or chronic obstructive pulmonary disorder	54 (17.9)	7 (38.9)	47 (16.5)	3.21 (1.13-8.58)	0.022	3.22 (1.11-8.92)	0.0254
Brain injury	57 (18.9)	5 (27.8)	52 (18.3)	1.72 (0.53-4.77)	0.3246		
Malignancy	39 (12.9)	3 (16.7)	36 (12.7)	1.38 (0.31-4.43)	0.6258		
Obesity	18 (6.0)	1 (5.6)	17 (6.0)	0.92 (0.05-4.94)	0.9404		
Carbapenem exposure	89 (29.5)	6 (33.3)	83 (29.2)	1.21 (0.41-3.23)	0.7112		
Amikacin exposure	36 (11.9)	3 (16.7)	33 (11.6)	1.52 (0.34-4.92)	0.5244		
Gentamicin exposure	10 (3.3)	0 (0.0)	10 (3.5)	0.00 (NA-Infinity)	0.9905		
Tobramycin exposure	17 (5.6)	1 (5.6)	16 (5.6)	0.99 (0.05-5.30)	0.9889		
Aztreonam exposure	10 (3.3)	0 (0.0)	10 (3.5)	0.00 (NA-Infinity)	0.9905		
Sulfamethoxazole-trimethoprim exposure	10 (3.3)	1 (5.6)	9 (3.2)	1.80 (0.09-10.42)	0.5883		
Cephalosporin exposure	70 (23.2)	7 (38.9)	63 (22.2)	2.23 (0.79-5.91)	0.1112	2.66 (0.92-7.36)	0.0606
Fluoroquinolone exposure	54 (17.9)	2 (11.1)	52 (18.3)	0.56 (0.09-2.04)	0.4456		
Daptomycin exposure	11 (3.6)	2 (11.1)	9 (3.2)	3.82 (0.55-16.44)	0.1034		
Metronidazole exposure	57 (18.9)	5 (27.8)	52 (18.3)	1.72 (0.53-4.77)	0.3246		
Linezolid exposure	46 (15.2)	4 (22.2)	42 (14.8)	1.65 (0.45-4.85)	0.399		
Polymyxin and/or colistin exposure	33 (10.9)	1 (5.6)	32 (11.3)	0.46 (0.03-2.38)	0.4619		
Tigecycline exposure	38 (12.6)	2 (11.1)	36 (12.7)	0.86 (0.13-3.20)	0.8462		
Vancomycin exposure	130 (43.0)	8 (44.4)	122 (43.0)	1.06 (0.39-2.77)	0.9017		
Piperacillin/Tazobactam exposure	42 (13.9)	2 (11.1)	40 (14.1)	0.76 (0.12-2.82)	0.7244		

Abbreviations: aOR, adjusted odds ratio; CI, confidence interval; OR, odds ratio.

Supplementary Figure 1. Cross-resistance profile of carbapenem-resistant *Klebsiella pneumoniae* ST258 isolates

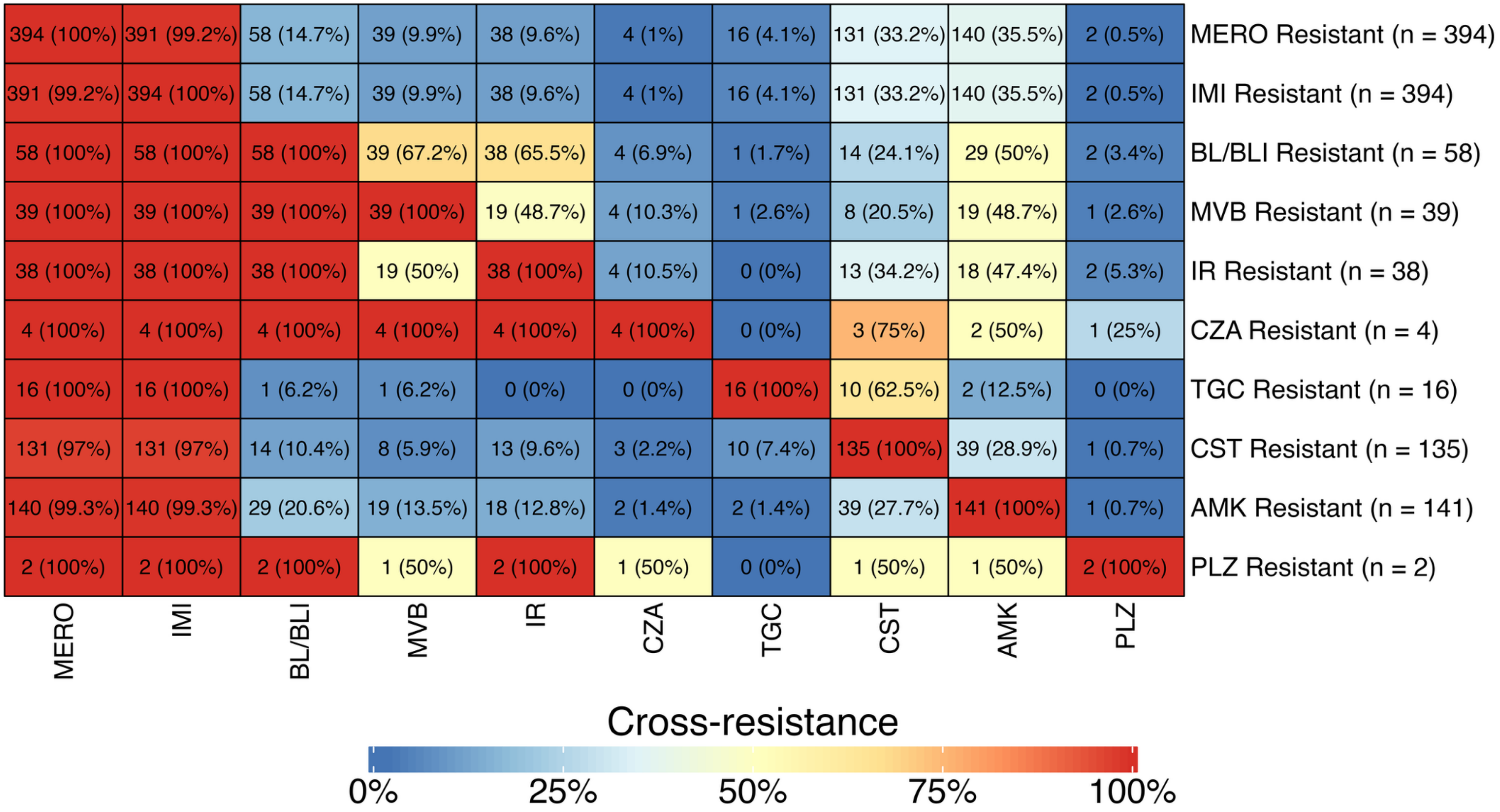

The cross-resistance profile of 412 carbapenem-resistant *Klebsiella pneumoniae* sequence type 258 strains. This heatmap shows the number of resistant isolates (rows) that also exhibit resistance to other antibiotics (columns). Cross-resistance was evaluated for the following antibiotics: amikacin, colistin, ceftazidime-avibactam, imipenem, imipenem-relebactam, meropenem, meropenem-vaborbactam, plazomicin, and tigecycline. Resistance was defined as a minimum inhibitory concentration within intermediate or resistant categories. Resistance to β-lactam/β-lactamase inhibitor combinations was defined as having resistance to imipenem-relebactam and/or meropenem-vaborbactam. **Abbreviations:** AMK, amikacin; BL/BLI, β-lactam/β-lactamase inhibitor; CST, colistin; CZA, ceftazidime-avibactam; IMI, imipenem; IR, imipenem-relebactam; MERO, meropenem, MVB, meropenem-vaborbactam; PLZ, plazomicin; TGC, tigecycline.

Supplementary Figure 2. Spearman's rank correlation coefficients between the minimum inhibitory concentration of last-line and novel agents

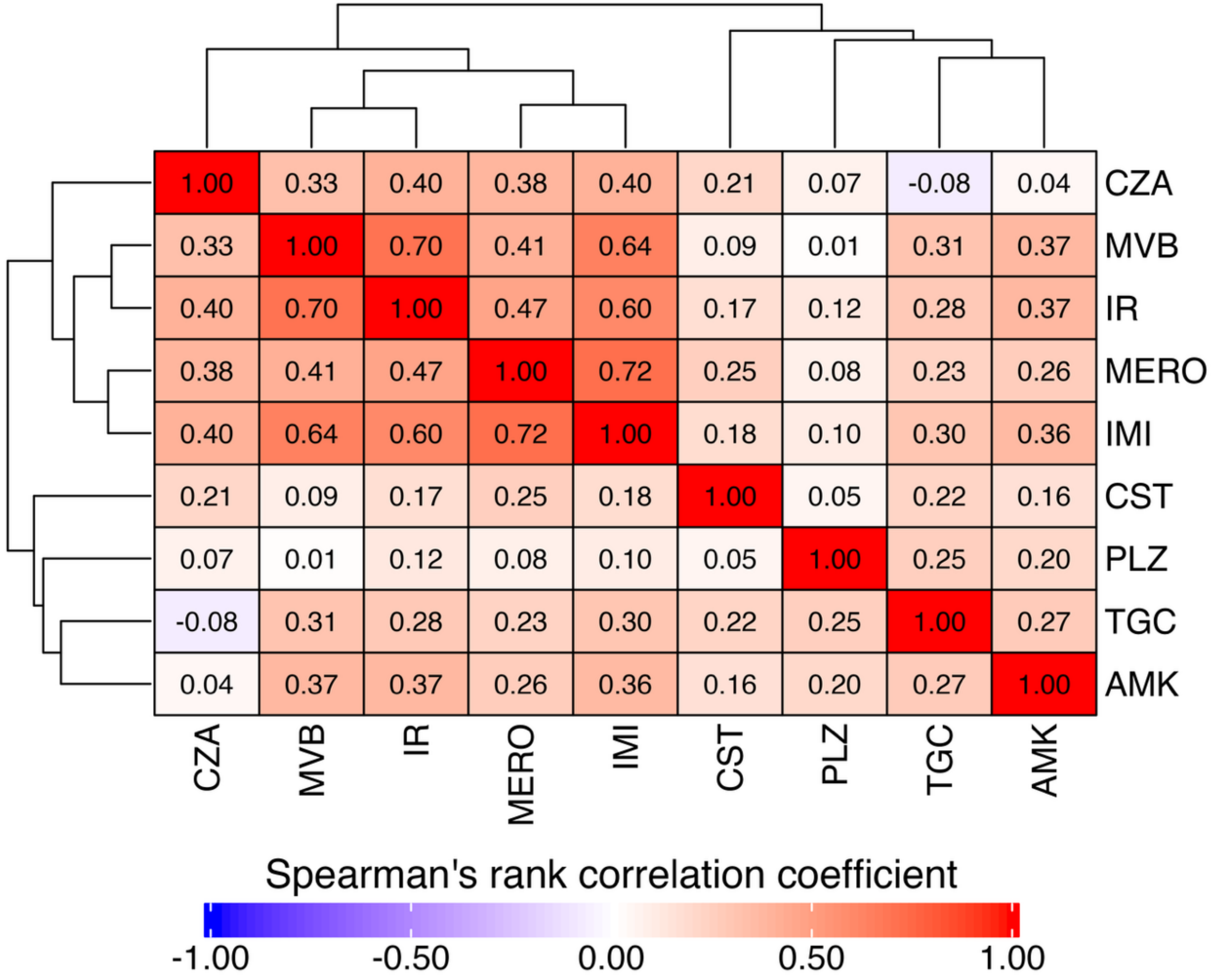

Spearman's rank correlation coefficient between minimum-inhibitory concentrations of last-line and novel agents for 412 carbapenem-resistant *Klebsiella pneumoniae* sequence type 258 strains. The following antibiotics were evaluated: amikacin, colistin, ceftazidime-avibactam, imipenem, imipenem-relebactam, meropenem, meropenem-vaborbactam, plazomicin, and tigecycline. **Abbreviations:** AMK, amikacin; CST, colistin; CZA, ceftazidime-avibactam; IMI, imipenem; IR, imipenem-relebactam; MERO, meropenem, MVB, meropenem-vaborbactam; PLZ, plazomicin; TGC, tigecycline.

Supplementary Figure 3. Clade-specific differences in non-carbapenemase mechanisms of carbapenem resistance

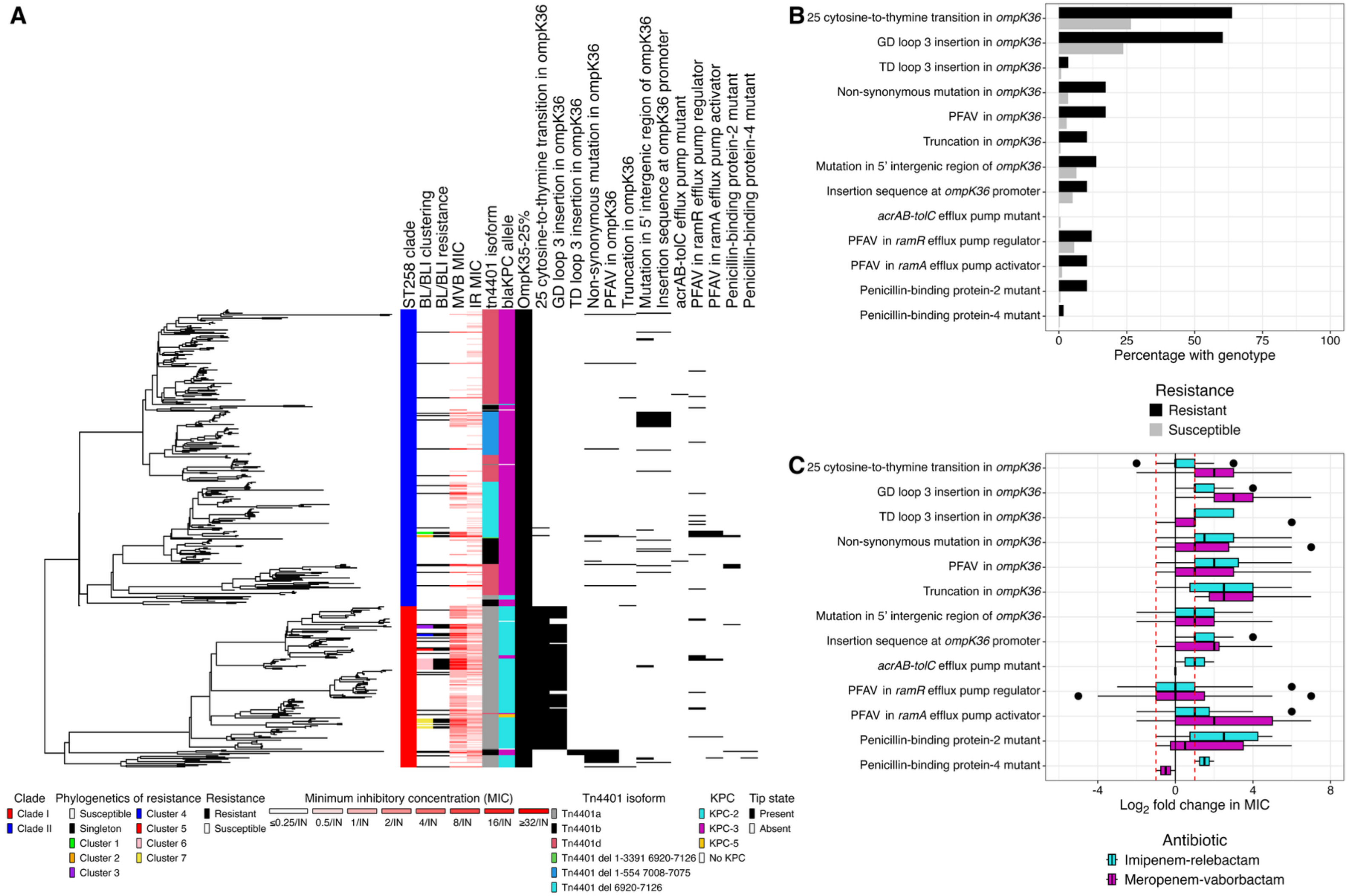

(**a**) Mechanisms of carbapenem resistance were overlaid across the sequence type 258 phylogeny. (**b**) The frequency of genotypes in β-lactam/β-lactamase inhibitor combination susceptible and resistant isolates. (**c**) The log_2 fold change in imipenem-relebactam and meropenem-vaborbactam minimum inhibitory concentration for isolates with the genotype relative to their nearest phylogenetic neighbor without the genotype. **Abbreviations:** BL/BLI, β-lactam/β-lactamase inhibitor; IR, imipenem-relebactam; MVB, meropenem-vaborbactam; PFAV, putative function-altering variant.

Supplementary Figure 4. All significant genome-wide association study hits for β-lactam/β-lactamase inhibitor resistance

A

Significant hit	Gene	Product	Significant tests	Significant models	Phenotypes	Nearest neighbor criterion
KPNIH1_RS02940		hypothetical protein	Continuous	All variants & Non-synonymous	MVB	Pass
KPNIH1_RS18100		MdtA/MuxA family multidrug efflux RND transporter periplasmic adaptor subunit	Continuous	Putative function-altering variants	IR	Pass
Loop 3 insertion in *ompK36*	*ompK36*	porin OmpC	Continuous	Kleborate	MVB	Pass
GD loop 3 insertion in *ompK36*	*ompK36*	porin OmpC	Continuous	Kleborate	MVB	Pass
PFAV in *ompK36*	*ompK36*	porin OmpC	All tests	All variant models	BL/BLI & IR & MVB	Pass
Truncation of *ompK36*	*ompK36*	porin OmpC	All tests	Kleborate	BL/BLI & IR & MVB	Pass
SHV-11		Broad-spectrum β-lactamase	Synchronous	Kleborate	BL/BLI	Fail
TEM-1D.v1^		Broad-spectrum β-lactamase	Synchronous	Kleborate	BL/BLI	Fail
AA018 *bla*$_{KPC}$-containing plasmid		*bla*$_{KPC}$-containing plasmid cluster	Synchronous	*bla*$_{KPC}$-containing plasmids	BL/BLI	Pass
AA552 *bla*$_{KPC}$-containing plasmid		*bla*$_{KPC}$-containing plasmid cluster	PhyC & Synchronous	*bla*$_{KPC}$-containing plasmids	BL/BLI	Pass

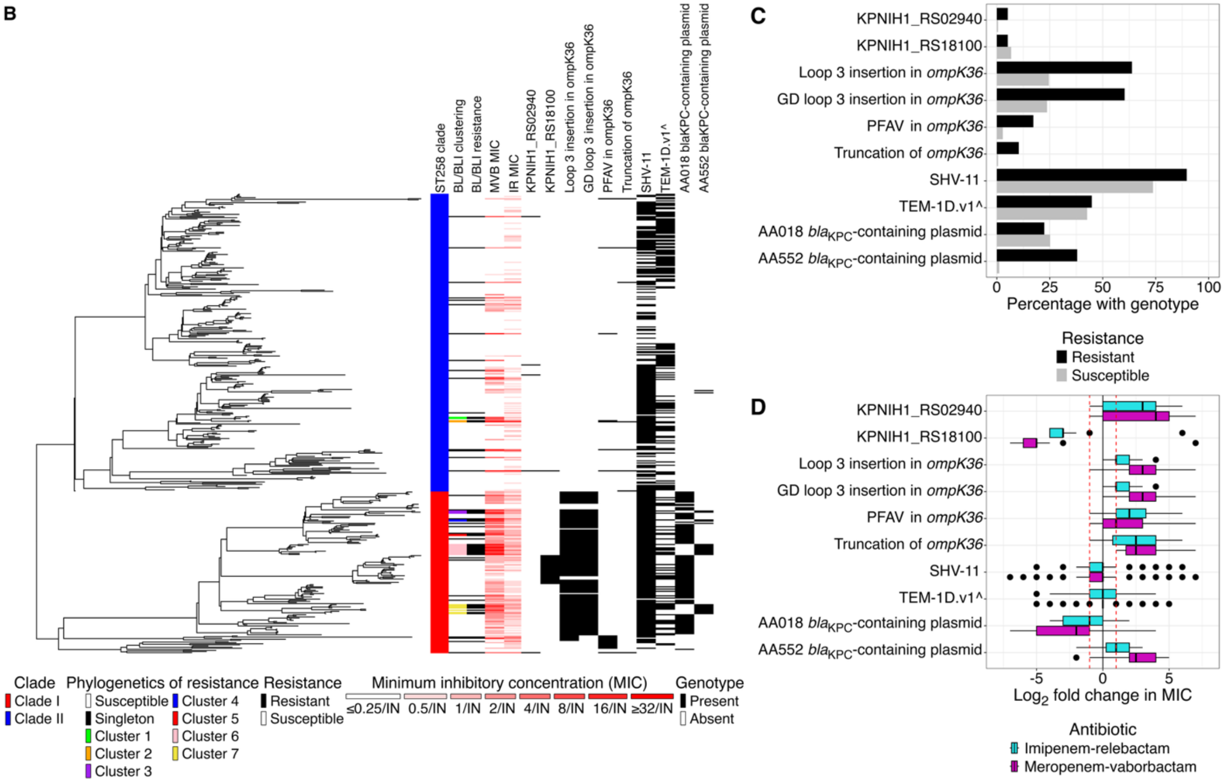

Convergence-based genome-wide association study was performed on $\log_2$ minimum inhibitory concentration and resistance to β-lactam/β-lactamase inhibitor combinations using hogwash v.1.2.6. Hits that passed the nearest neighbor criterion (median $\log_2$ fold change in MIC > |1|) were eligible for downstream analysis. (**a**) Information about each significant genome-wide association study hit. (**b**) The significant hits are overlaid on the phylogenetic tree. **c** The frequency of genotypes in β-lactam/β-lactamase inhibitor combination susceptible and resistant isolates. (**d**) The $\log_2$ fold change in imipenem-relebactam and meropenem-vaborbactam minimum inhibitory concentration for isolates with the genotype relative to their nearest phylogenetic neighbor. **Abbreviations:** BL/BLI, β-lactam/β-lactamase inhibitor; IR, imipenem-relebactam; MVB, meropenem-vaborbactam; PFAV, putative function-altering variant.

Supplementary Figure 5. Differences in bla_{KPC} plasmid backbone contribute to elevated bla_{KPC} copy number and the spread of resistance in clade I

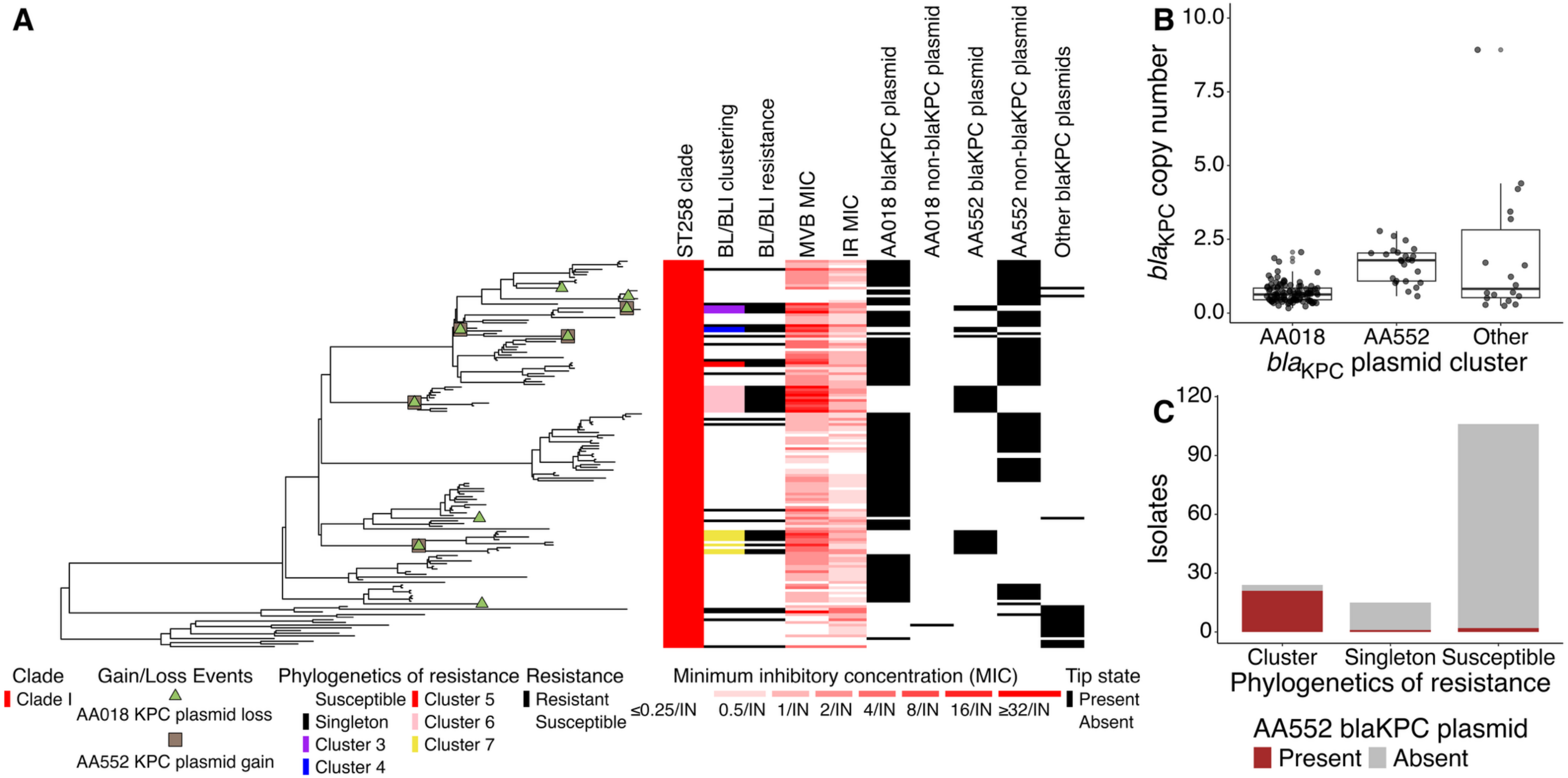

(**a**) The presence of dominant *Klebsiella* pneumoniae carbapenemase (bla_{KPC})-containing plasmids across clade I. The emergence of resistance and select resistance-associated genotypes is overlaid on ancestral nodes. (**b**) Differences in bla_{KPC} copy number across bla_{KPC} plasmids. (**c**) The frequency of bla_{KPC}-containing AA552 plasmids in β-lactam/β-lactamase inhibitor resistant clusters, singletons, and susceptible isolates. **Abbreviations:** BL/BLI, β-lactam/β-lactamase inhibitor; IR, imipenem-relebactam; MVB, meropenem-vaborbactam; PFAV, putative function-altering variant.

Supplementary Figure 6. Evidence of the *tn4401 bla*$_{KPC}$ transposon hopping from AA018 plasmid to the AA552 plasmid

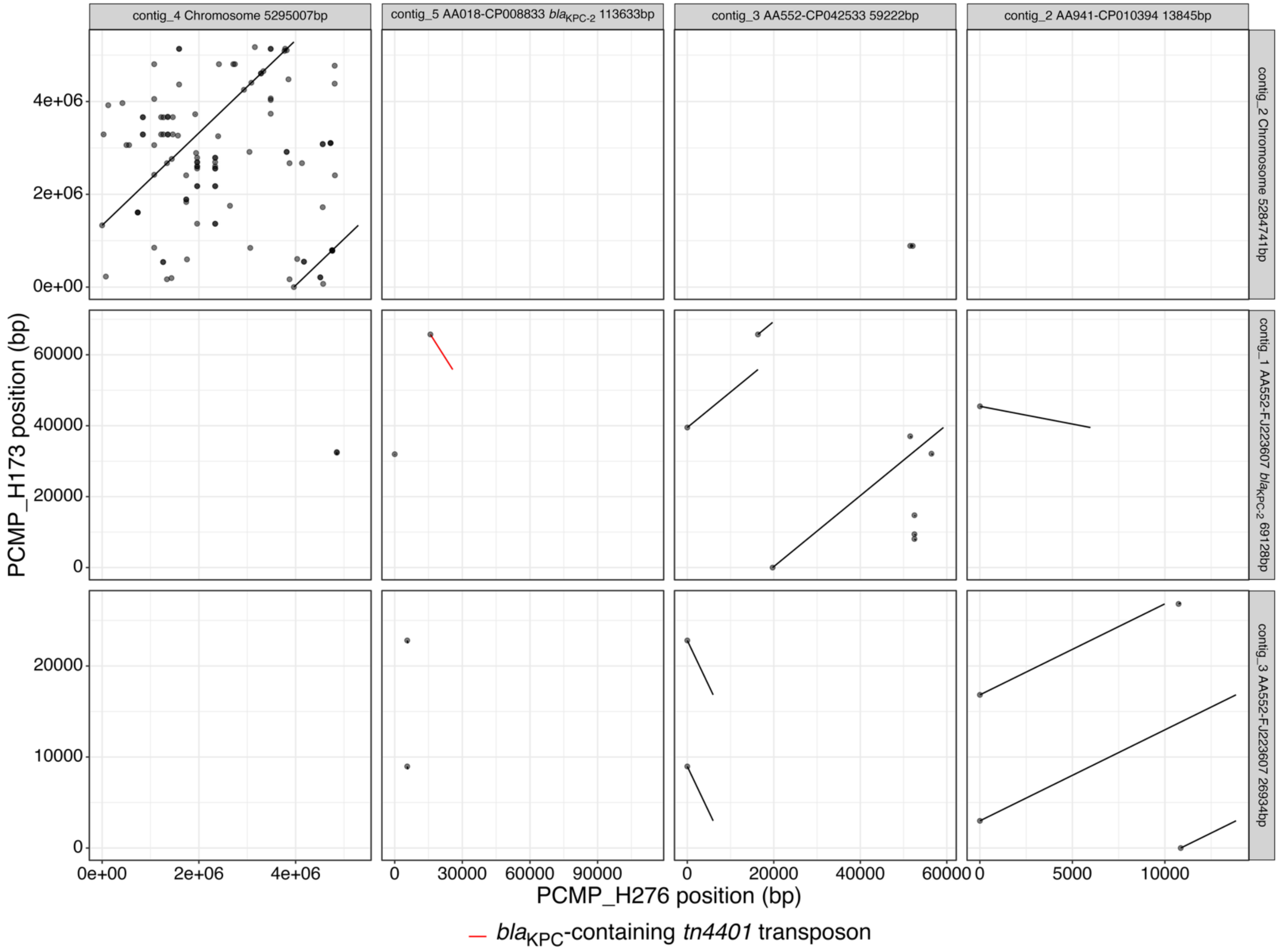

Long-read sequencing was performed on a representative clade I isolate with *Klebsiella pneumoniae* carbapenemase (*bla*$_{KPC}$)-containing AA552 plasmid and a closely related isolate with a *bla*$_{KPC}$-containing AA018 plasmid. Grids were named using the isolate's contig name, Mob-Suite plasmid calls (primary cluster – mash nearest neighbor), the presence of the *bla*$_{KPC}$ gene, and contig size. The presence of the bla$_{KPC}$ transposon, *tn4401*, was labeled in red.

Supplementary Figure 7. Performance statistics for resistance and our genotype panels

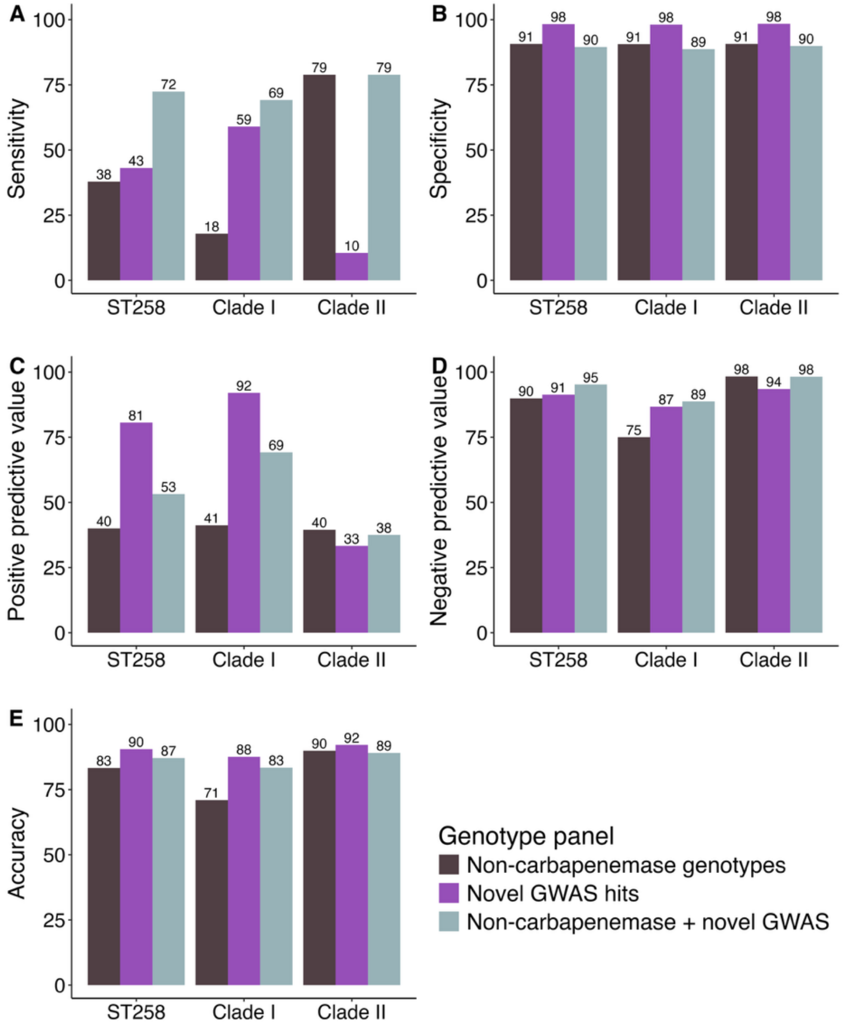

The sensitivity (**a**), specificity (**b**), positive predictive value (**c**), negative predictive value (**d**), and accuracy (**e**) of our genotype panels for resistance to β-lactam/β-lactamase inhibitor combinations. The following genotypes were considered in our known carbapenem resistance-associated panel: truncations and putative function-altering variants in *ompK36*, insertion sequences near the *ompK36* promoter, putative function-altering variants in *ramA* efflux pump activator, and non-synonymous mutations in PBPs. The following novel hits were identified in our genome-wide association study: *Klebsiella pneumoniae* carbapenemase (*bla*$_{KPC}$)-containing AA552 plasmid and the hypothetical protein, KPNIH1_RS02940. **Abbreviations:** BL/BLI, β-lactam/β-lactamase inhibitor; IR, imipenem-relebactam; MVB, meropenem-vaborbactam; PFAV, putative function-altering variant.

Supplementary Figure 8. Differential potential for emergence and spread of β-lactam/β-lactamase inhibitor resistance-associated genotypes

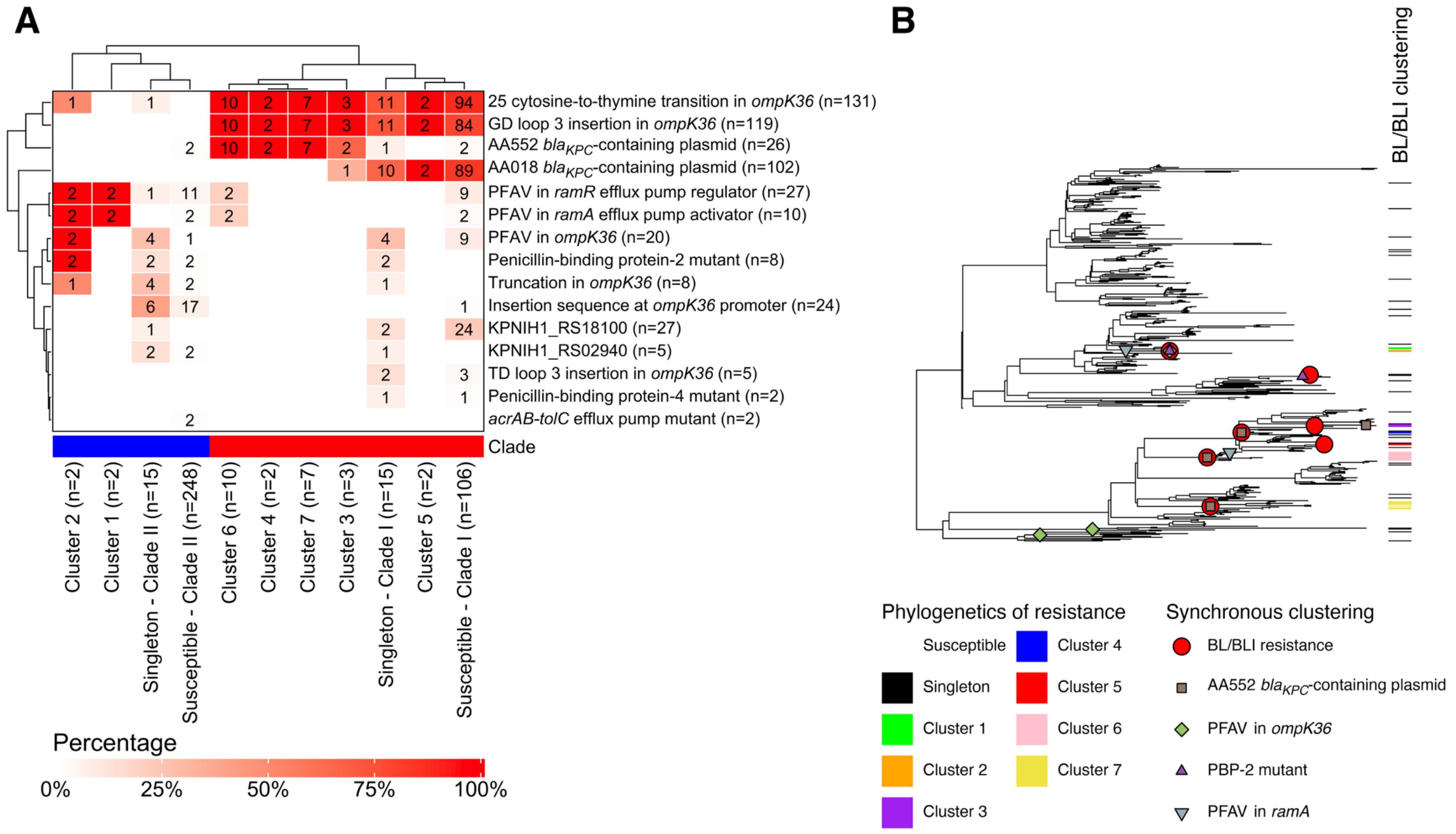

(**a**) The frequency of genotypes in phylogenetic singletons of β-lactam/β-lactamase inhibitor (BL/BLI) resistance, clusters of BL/BLI resistance, and susceptible isolates. (**b**). The synchronous clustering of BL/BLI resistance and resistance genotypes is overlaid on ancestral nodes. **Abbreviations:** BL/BLI, β-lactam/β-lactamase inhibitor; PFAV, putative function-altering variants; PBP, penicillin-binding protein.

Supplementary Figure 9. Percentage of isolates with a core genome variant

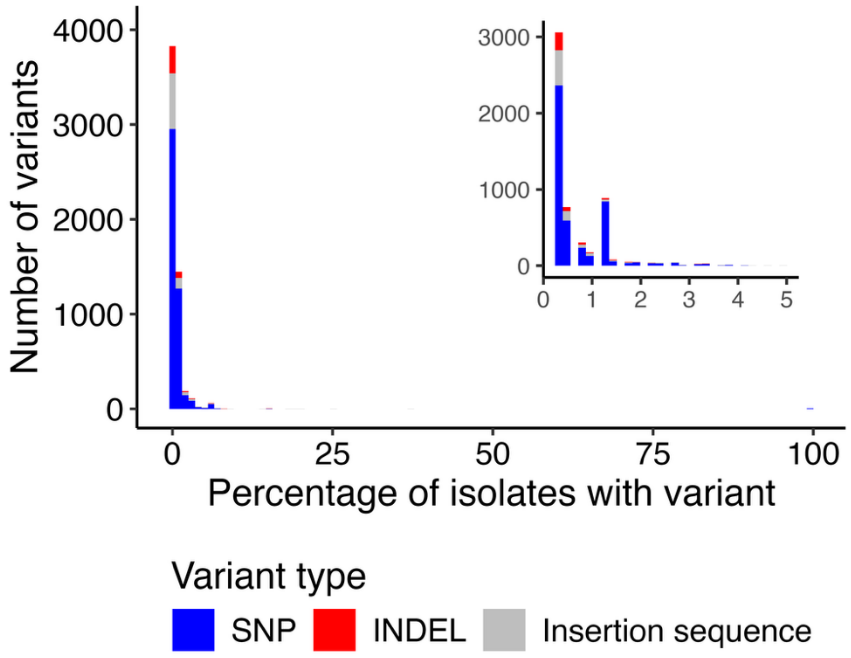

The percentage of isolates with considered variants: single-nucleotide polymorphisms, insertions, deletions, and insertion sequences. The figure insert highlights the distribution of variants that were found in less than five percent. **Abbreviations:** INDEL, insertion or deletion; SNP, single-nucleotide polymorphism
